# The characteristic and prognostic role of blood inflammatory markers in patients with Huntington’s disease from China

**DOI:** 10.3389/fneur.2024.1374365

**Published:** 2024-03-26

**Authors:** Jie-Qiang Xia, Yang-Fan Cheng, Si-Rui Zhang, Yuan-Zheng Ma, Jia-Jia Fu, Tian-Mi Yang, Ling-Yu Zhang, Jean-Marc Burgunder, Hui-Fang Shang

**Affiliations:** ^1^Department of Neurology, Laboratory of Neurodegenerative Disorders, Rare Disease Center, West China Hospital, Sichuan University, Chengdu, Sichuan, China; ^2^Department of Neurology, The First People's Hospital of Shuangliu District, Chengdu, China; ^3^Department of Neurology, Swiss Huntington's Disease Centre, Siloah, University of Bern, Bern, Switzerland

**Keywords:** Huntington’s disease, peripheral inflammatory, progression, prognosis, biomarkers

## Abstract

**Objectives:**

This study aims to elucidate the role of peripheral inflammation in Huntington’s disease (HD) by examining the correlation of peripheral inflammatory markers with clinical manifestations and disease prognosis.

**Methods:**

This investigation involved 92 HD patients and 92 matched healthy controls (HCs). We quantified various peripheral inflammatory markers and calculated their derived metrics including neutrophil-to-lymphocyte ratio (NLR), platelet-to-lymphocyte ratio (PLR), lymphocyte-to-monocyte ratio (LMR), and systemic immune-inflammation index (SII). Clinical assessments spanning cognitive, motor, and disease severity were administered. Comparative analysis of inflammatory markers and clinical correlations between HD and controls was performed. Kaplan–Meier survival analysis and Cox regression model were used to assess the effect of inflammatory markers on survival.

**Results:**

The study revealed that HD patients had significantly reduced lymphocyte counts, and LMR. Conversely, NLR, PLR, and SII were elevated compared to HCs. Lymphocyte levels inversely correlated with the age of onset and monocyte levels inversely correlated with the UHDRS-total functional capacity (TFC) scores. After adjusting for age, sex, and CAG repeat length, lymphocyte count, NLR, PLR, and SII were significantly correlated with the progression rate of TFC scores. Elevated levels of white blood cells and monocytes were associated with an increased risk of disability and mortality in the HD cohort.

**Conclusion:**

Our findings indicate that HD patients display a distinct peripheral inflammatory profile with increased NLR, PLR, and SII levels compared to HCs. The peripheral inflammation appears to be linked with accelerated disease progression and decreased survival in HD.

## 1 Introduction

Huntington’s disease (HD) is a hereditary neurodegenerative condition characterized by an autosomal dominant inheritance pattern. It is characterized by chorea, cognitive decline, and psychiatric and behavioral changes (1). An abnormal amplification of CAG causes HD repeats in exon 1 of the Huntingtin (*HTT*) gene, located on chromosome 4 (1). It usually begins to manifest in mid-adulthood and lasts for about 15–20 years (2, 3). To date, there are no effective disease-modifying therapies, imposing a heavy economic burden on families and society which increases as the disease progresses (4). Although the complete pathogenesis of HD is not yet fully understood, several potential mechanisms have been proposed, such as excitotoxicity, dopaminergic imbalance, mitochondrial dysfunction, metabolic defects, disruption of proteostasis, transcriptional dysregulation, and neuroinflammation (5). Current evidence suggests that neuroinflammation is a strong component of HD pathogenesis, including triggering and amplifying degeneration (6, 7). Evidence suggests that the brain and the peripheral immune system interact through various pathways, either by the glymphatic pathway or by a blood–brain barrier disruption (8, 9). This process results in an increase in the number of blood cells in the circulation that are involved in inflammation and immune response (10).

Systemic inflammation can be assessed using various hematological markers in standard blood tests, including peripheral neutrophils, monocytes, lymphocytes, and platelets (11). Inflammatory markers including the neutrophil-to-lymphocyte ratio (NLR), platelet-to-lymphocyte ratio (PLR), lymphocyte-to-monocyte ratio (LMR), and systemic immune-inflammation index (SII), have been identified as emerging biomarkers that reflect the balance between innate immunity and adaptive immunity (11). These markers are used in prognosticating various conditions, such as cancer (12, 13), cardiovascular diseases (14), acute ischemic strokes (15, 16), and infectious diseases (17). The elevated NLR has been recognized as a noteworthy independent predictor for Parkinson’s disease (PD) (18), Alzheimer’s disease (AD) (19, 20), and progressive supranuclear palsy (PSP) (21). Studies have shown that NLR can be used as a prognosis biomarker for amyotrophic lateral sclerosis (ALS) (22–24), and multiple system atrophy (MSA) (25). Increased SII is associated with an increased risk of developing ALS (11).

However, no report exists on the association between inflammatory composite indicators (including NLR, PLR, LMR, and SII) and disease severity in HD and there is also no comparative analysis of these indicators in HD progression. We aim to evaluate the systemic inflammation status by analyzing parameters derived from a complete blood count (CBC) in patients with HD, and to compare these parameters with those of healthy controls (HCs). Furthermore, we aim to investigate potential associations between the clinical characteristics of HD patients and inflammatory indicators. Ultimately, our goal is to explore the feasibility of utilizing these biomarkers as prognostic factors in HD.

## 2 Methods

### 2.1 Study design and participants

In this study, 92 individuals with gene-confirmed HD were recruited between March 2009 and November 2023 in the Department of Neurology at West China Hospital of Sichuan University (Chengdu, China). The participants in this study were from the same cohort as the previous study (3, 26). The study received approval from the Institutional Ethics Committee of West China Hospital, Sichuan University, China. Each participant diagnosed with HD in the study had a CAG repeat length more than 39, which was confirmed through genetic testing, and exhibited motor disturbances as defined by a diagnostic confidence score of 4 on the Unified Huntington’s Disease Rating Scale (UHDRS) total motor score (TMS) (27, 28). Exclusion criteria included individuals with chronic inflammatory disease, infectious illness within 3 days prior to the blood test, chronic heart disease, liver cirrhosis, autoimmune diseases, and the use of vitamin or antioxidant supplements. Ninety two HC were recruited and matched for age, sex, and body mass index (BMI). Neurologists (JQX, YFC, and SRZ) through telephone or in-person interviews until November 2023. One patient was lost to follow-up.

### 2.2 Demographic and clinical data collection

All participants underwent face-to-face interviews with experienced neurologists to collect demographic characteristics, including age, gender, height, weight, education, family history, smoking and alcohol consumption habits, as well as age and symptom onset, disease duration, CAG repeat length, comorbidities, and treatments at the baseline. BMI was computed by dividing body weight (kg) by the squared of the height (m^2^). The age of onset refers to the point at which symptoms related to movement, cognitive, or psychiatric impairments first begin to appear. The age of motor symptom onset (AAO) is defined as when movement disorder begins to manifest. The age at diagnosis is determined by subtracting the date of birth from the date when the gene test was conducted. Clinical severity was assessed by UHDRS (29). Motor function was assessed by TMS. Daily functional performance was assessed by UHDRS-total functional capacity (TFC). Cognitive function was evaluated using the mini-mental state examination (MMSE), Symbol Digit Modality Test (SDMT), Stroop word reading test (SWR), Stroop color naming test (SCN), Stroop interference test (SI), and Trail Making Test (TMT) A and B. Psychiatric symptoms were assessed by the Hamilton Depression Scale (HAMD), Hamilton Anxiety Scale (HAMA), Beck Depression Inventory (BDI), and the short version of Problem-Behavior Assessment (PBA-s). Furthermore, the rate of disease progression was presented as TFC score decline rate [TFC progression rate = (Baseline (TFC)-last following-up (TFC))/Time of following-up]. This was determined by calculating the number of points lost annually from diagnosis to last follow-up. Stages of HD were classified based on UHDRS-TFC score: early (Stage I, TFC 11–13), early-mid (Stage II, TFC 7–10), and advanced disease states (Stage III-V, TFC 0–6) (30). Based on the long survival time of patients with HD, we defined the loss-of-function state as death or TFC score of 0–2. The loss-of-function state was defined as the period from the date of disease diagnosis to death for a deceased patient, or from disease diagnosis in surviving patients to the time when TFC score reached 0–2 at the last follow-up. Death information was collected from family reports.

### 2.3 Laboratory data collection

All participants in the study underwent routine blood tests upon admission to measure the count of white blood cells (WBC), including neutrophils, lymphocytes, monocytes, eosinophils, and basophils, as well as the count of red blood cells (RBC), hemoglobin levels, and platelets. Inflammatory markers (including NLR, PLR, LMR, and SII) were calculated. The NLR was calculated as the neutrophil count/lymphocyte count. PLR was calculated as the platelet count/lymphocyte count. LMR was calculated as the lymphocyte count/monocyte count. SII was calculated as the (neutrophil count*platelet count)/lymphocyte count (11). The increased levels of neutrophils, monocytes, NLR, PLR, and SII indicate higher innate immunity, while increased levels of lymphocytes and LMR indicate higher adaptive immunity (31).

### 2.4 Statistical analysis

Demographic and clinical data were analyzed with SPSS (version 20.0, IBM, United States), R studio, and Prism GraphPad version 9. *p* values < 0.05 were considered statistically significant. The data was presented using the following conventions: mean ± standard deviation (SD) for continuous data and frequency and percentage for categorical variables. To compare continuous variables between the HD and HC groups, either the Mann–Whitney U test or the Student’s *t*-test was utilized. Conversely, the chi-square test was employed to compare categorical variables. The age groups (<40, 40–50, and >50) were chosen based on the distribution of our study sample, which primarily included adults, with fewer adolescents and older individuals. There were less than five participants with a BMI < 18 or a BMI > 25. Therefore, participants were stratified based on their mean BMI and age of onset (<40, 40–50, >50 years) to ensure equal representation across different groups. Next, analyses were performed between groups of different genders (male and female patients), age of onset, disease stages, and mean BMI at recruitment (<20.73 and ≥20.73). To ensure a robust statistical approach, we have employed Analysis of Covariance (ANCOVA) to adjust for potential confounders such as sex, BMI, age of onset, as well as age and disease severity or CAG repeat length. These adjustments allow for a more accurate interpretation of the influence of age on the disease parameters studied. Either Pearson’s correlation test or Spearman’s correlation test was performed to assess the association between inflammatory markers and clinical characteristics, as well as disease progression. We employed a two-step regression analysis to evaluate the correlation between clinical characteristics and inflammatory indicators. Our first step involved conducting Pearson’s or Spearman’s correlation analysis for each inflammatory indicator. In the second step, we performed multivariate linear regression analyses by adjusting for age, sex, and CAG repeat length and the variables with a *p*-value < 0.05 from the previous step. We provided standardized β coefficients for the variables associated with each inflammatory indicator. The patients were categorized into three subgroups based on LMR, NLR, PLR, and SII tertiles. Similarly, patients were divided into three groups according to WBC and monocyte tertiles as follows: high WBC group (Group 1) [WBC counts ≥ 6.07*10^9^/L (*n* = 31)], medium WBC group (Group 2) [WBC counts > 4.84*10^9^/L but < 6.07*10^9^/L (*n* = 30)], and low WBC group (Group 3) [WBC counts ≤ 4.84*10^9^/L (*n* = 31)]; high monocyte group (Group1) [monocyte counts ≥0.39*10^9^/L (*n* = 31)], medium monocyte group (Group 2)[monocyte counts > 0.39*10^9^/L but <0.28*10^9^/L (*n* = 30)], and low monocyte group (Group 3) [monocyte counts ≤0.28*10^9^/L (*n* = 31)] We employed the Kaplan–Meier survival analysis and Breslow test to assess the prognostic significance of inflammatory indicators for the occurrence of loss-of-function. Furthermore, we executed univariate and multivariate survival analyses using the Cox proportional hazards regression model, adjusting for age, sex, and CAG repeat length with the multivariate Cox proportional hazards regression model.

## 3 Results

### 3.1 Baseline characteristics

Total of 92 HD patients with 38.04% males and 92 age, sex-matched HCs were enrolled in the study based on the inclusion and exclusion criteria. Baseline characteristics of the subjects are summarized in Table 1. The lack of TFC score at enrollment was excluded when analyzed by disease stage (*n* = 3). The participants were classified into 3 groups: Stage I (early HD, *n* = 21), Stage II (early-mid HD, *n* = 39), and Stage III-V (advanced HD, *n* = 29). The mean age of patients and age of onset were 54.27 ± 11.36 and 42.67 ± 10.04 years, respectively. The mean disease duration at baseline was 5.41 ± 3.93 years. The average length of CAG repeats was 44 (42–46). No significant differences were found in the age, sex, smoking, drinking, and BMI values between the HD patients and HCs (all *p* > 0.05).

**Table 1:** Demographic characteristics of patients with Huntington’s disease (HD) and healthy controls (HCs).

	HC (*n* = 92)	HD (*n* = 92)	*p*-value	HD subgroups			*p*-value
				Stage I (*n* = 21)	Stage II (*n* = 39)	Stage III-V (*n* = 29)	
Age (years)	54.76 ± 4.49	54.27 ± 11.36	0.791	53.91 ± 10.72	54.22 ± 11.59	56.98 ± 10.93	0.525
Sex (male, %)	39.10% (36/92)	38.04% (35/92)	1.0	33.33% (7/21)	38.40% (15/39)	41.30% (12/29)	0.845
BMI (kg/m^2^)	21.69 ± 1.462	20.93 ± 2.64	0.840	21.92 ± 1.66	20.63 ± 2.220	21.47 ± 3.48	0.470
Education (years)	/	9.13 ± 4.45	/	9.62 ± 4.61	9.59 ± 4.56	7.64 ± 4.65	0.184
Smoking (%)	26.09% (24/92)	23.81% (10/42)	0.865	0	42.11% (8/19)	12.50% (2/16)	<0.001*
Drinking (%)	31.52% (29/92)	26.19% (11/42)	0.515	0	31.58% (6/19)	31.25% (5/16)	0.011*
**Comorbidity**							
Hypertension (%)	/	0% (0/42)		0	0	0	1
Diabetes mellitus (%)	/	7.14% (3/42)		0	10.00% (2/20)	6.25% (1/16)	0.319
Hyperlipidemia (%)	/	2.38% (1/42)		0	5.00% (1/20)	0	0.269

BMI, body mass index; HC, healthy control; HD, Huntington’s disease.

### 3.2 Inflammatory indicators in HD patients and HCs

The hematological data for patients with HD and HCs are presented in Table 2. HD patients exhibited lower levels of RBC count (4.50 ± 0.48 vs. 4.68 ± 0.40 10^12^/L, *p* = 0.007), hemoglobin (134.0 ± 17.20 vs.142.50 ± 14.86 g/L, *p* = 0.001), lymphocyte (1.67 ± 0.47 vs.1.90 ± 0.58 10^9^/L, *p* = 0.005), basophilia (0.02 ± 0.01 vs.0.03 ± 0.02 10^9^/L, *p* = 0.002), and LMR (5.13 ± 1.68 vs. 5.80 ± 2.11, *p* = 0.018) when compared to those of HCs. On the other hand, these patients had higher NLR (2.15 ± 0.90 vs. 1.71 ± 0.58, *p* = 0.0002), PLR (127.20 ± 51.59 vs. 111.0 ± 44.39, *p* = 0.024) and SII (420.40 ± 195.00 vs. 345.8 ± 1 61.8, *p* = 0.006) than those of HCs. The absolute leukocyte count, neutrophils, monocytes, and platelets were not significantly different between HD patients and HCs.

**Table 2:** Peripheral inflammatory indicators in patients with Huntington’s disease (HD) and healthy controls (HCs).

	HC (*n* = 92)	HD (*n* = 92)	*p*-value	HD Subgroups			*p*-value
				Stage I (*n* = 21)	Stage II (*n* = 39)	Stage III–V (*n* = 29)	
RBCs (10^12^/L)	4.68 ± 0.40	4.50 ± 0.48	0.007*	4.52 ± 0.56	4.61 ± 0.48	4.34 ± 0.40	0.079
Hemoglobin (g/L)	142.50 ± 14.86	134.00 ± 17.20	0.001*	133.60 ± 18.43	138.00 ± 15.47	128.20 ± 17.98	0.070
Platelet (10^9^/L)	197.00 ± 55.48	202.90 ± 72.73	0.532	191.60 ± 47.63	197.00 ± 59.01	210.00 ± 97.60	0.627
WBCs (10^9^/L)	5.67 ± 1.37	5.45 ± 1.30	0.269	5.03 ± 1.01	5.80 ± 1.46	5.24 ± 1.16	0.060
Neutrophils (10^9^/L)	3.17 ± 0.91	3.23 ± 0.99	0.665	3.02 ± 0.71	3.49 ± 1.11	3.02 ± 0.93	0.096
Lymphocytes (10^9^/L)	1.90 ± 0.58	1.67 ± 0.47	0.005*	1.55 ± 0.48	1.71 ± 0.47	1.67 ± 0.47	0.482
Monocyte (10^9^/L)	0.35 ± 0.12	0.35 ± 0.13	0.837	0.32 ± 0.12	0.36 ± 0.10	0.38 ± 0.16	0.225
Eosinophilia (10^9^/L)	0.11 ± 0.07	0.11 ± 0.07	0.747	0.10 ± 0.05	0.10 ± 0.07	0.11 ± 0.08	0.897
Basophilia (10^9^/L)	0.03 ± 0.02	0.02 ± 0.01	0.002*	0.02 ± 0.01	0.02 ± 0.01	0.02 ± 0.01	0.152
LMR	5.80 ± 2.11	5.13 ± 1.68	0.018*	5.26 ± 1.40	4.82 ± 1.47	5.10 ± 1.71	0.530
NLR	1.71 ± 0.58	2.15 ± 0.90	0.001*	2.07 ± 0.68	2.36 ± 1.01	1.94 ± 0.84	0.146
PLR	111.00 ± 44.39	127.20 ± 51.59	0.024*	131.00 ± 42.41	123.20 ± 48.02	128.30 ± 64.21	0.844
SII	345.80 ± 161.80	420.40 ± 195.00	0.005*	398.20 ± 168.40	436.80 ± 188.00	406.60 ± 221.00	0.717

HC, healthy control; LMR, lymphocyte-to-monocyte ratio; NLR, neutrophil-to-lymphocyte ratio; PLR, platelet-to-lymphocyte ratio; SII, systemic immune-inflammation index.

^*^p < 0.05.

### 3.3 Inflammatory indicators in HD patients across different sex, age of onset, stage, and BMI groups

**Figure 1:**
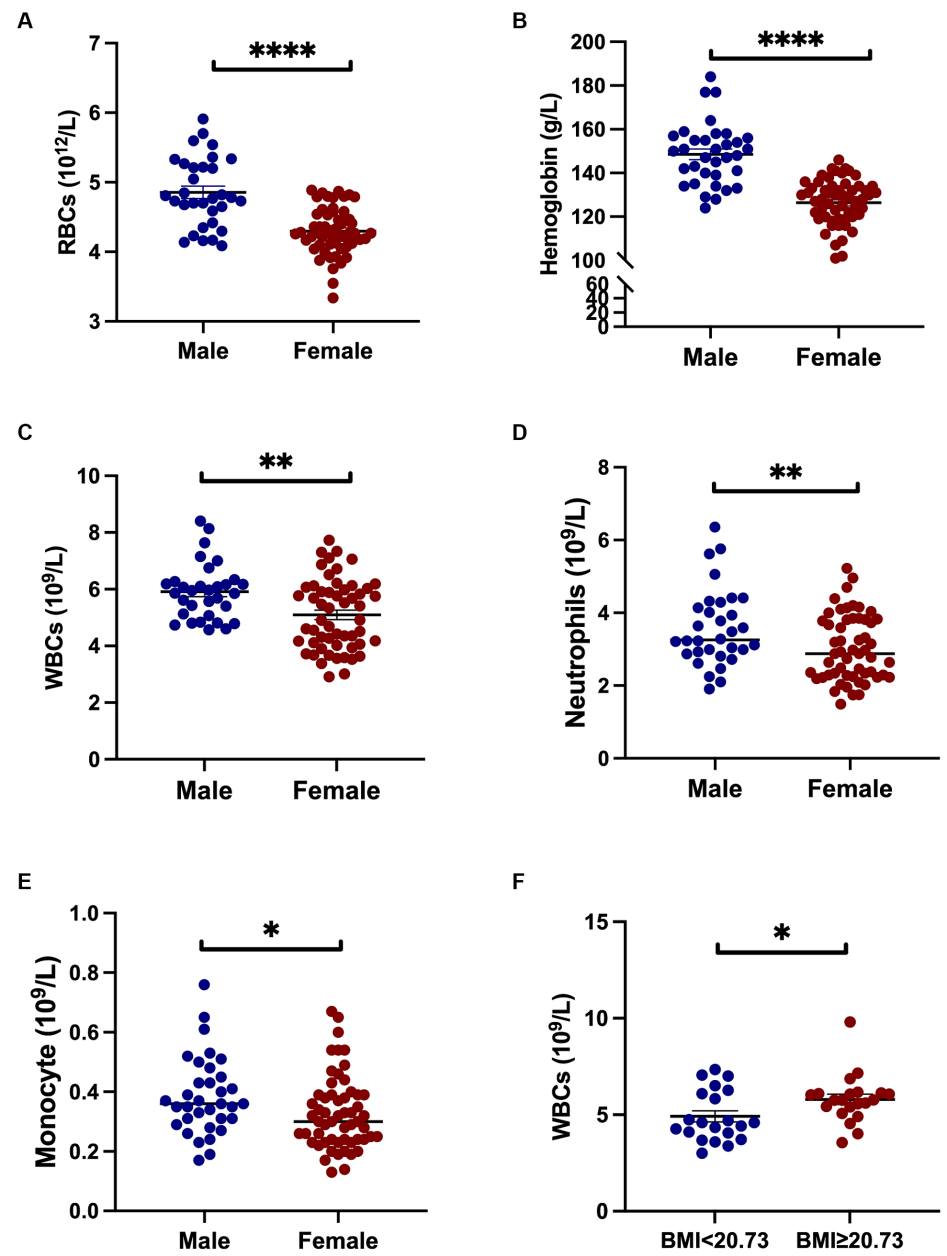
Inflammatory indicators in HD patients across sex and BMI groups. Male patients exhibited higher counts of RBCs, hemoglobin, WBCs, neutrophils, and monocytes compared to female patients **(A–E)**. An elevated BMI was associated with increased WBC counts relative to patients with lower BMI **(F)**. Specifically, *represents a *p*-value < 0.05; ** represents a *p*-value < 0.01; and **** represents a *p*-value < 0.0001.

**Figure 2:**
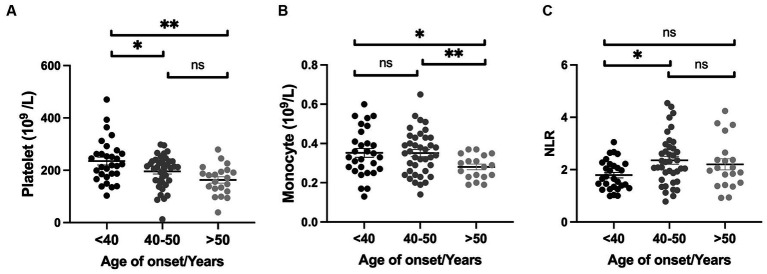
Inflammatory indicators in HD patients across age of onset. Platelet levels were elevated in patients with onset <40 years of age compared to those between 40 and 50 years and those with onset >50 years **(A)**. The level of monocytes in patients with onset <40 years and those aged 40–50 years were higher than in patients with onset >50 years **(B)**. The NLR level of patients aged 40–50 years of onset was higher compared to that of patients younger than 40 **(C)**. Specifically, * represents a *p*-value < 0.05; ** represents a *p*-value < 0.01.

Next, we performed subgroup analyses based on sex, age of onset, disease stages, and mean BMI to explore the differences in inflammatory indicators in HD patients. When HD patients were stratified by sex, we found that the levels of RBCs, hemoglobin, WBCs, neutrophils and monocytes were higher in males than in females (all *p* < 0.05) (Figures 1A–E). Compared with females, NLR in males tends to increase (*p* = 0.056). Male patients with HD were found to have lower hemoglobin levels (146.60 ± 18.17 vs. 154.9 ± 12.24 g/L, *p* = 0.047) and higher NLR (2.37 ± 1.16 vs. 1.84 ± 0.61, *p* = 0.020) when compared to healthy male individuals. On the other hand, female HD patients had lower levels of RBC count (4.30 ± 0.33 vs. 4.48 ± 0.26 10^12^/L, *p* = 0.002), hemoglobin (126.40 ± 11.04 vs. 135.20 ± 8.52 g/L, *p* < 0.0001), lymphocyte (1.61 ± 0.46 vs. 1.89 ± 0.59 10^9^/L, *p* = 0.005), higher NLR (2.01 ± 0.67 vs. 1.67 ± 0.61, *p* = 0.007), and SII (399.30 ± 147.50 vs. 327.40 ± 143.10, *p* = 0.011) than healthy female individuals. There were no significant differences in inflammatory indicators stratified by TFC stage. Platelet levels were higher in patients with age of onset <40 years old than those between 40 and 50 years and those with age of onset >50 years (Figure 2A). The level of monocytes in patients with age of onset <40 and 40–50 years old were higher than that in patients with age of onset >50 years (Figure 2B). The NLR level of patients with age of onset of aged 40–50 years was higher than that of patients with age of onset of younger than 40 (Figure 2C). Patients with high BMI had significantly higher WBC counts than those with low BMI (Figure 1F). In the HD group, no significant differences in the PLR, LMR, and SII were found according to sex, age of onset, disease severity (based on TFC score) and BMI. More details are shown in Supplementary Tables S1–S3.

### 3.4 Association of inflammatory indicators with clinical characteristics in HD patients

We further studied the correlation between the levels of inflammation indicators and clinical features using Pearson’s or Spearman’s correlation analysis. The detailed clinical characteristics of HD patients are shown in Supplementary Table S4. There was a significant correlation between NLR and age of onset (*r* = 0.225, *p* = 0.033), age of diagnosis (*r* = 0.209, *p* = 0.047), age of motor symptom onset (AAO) (*r* = 0.223, *p* = 0.035), SDMT (*r* = −0.278, *p* = 0.022). Lymphocytes negatively correlated with the age of onset (*r* = −0.274, *p* = 0.009) and AAO (*r* = −0.227, *p* = 0.032). PLR negatively correlated with age of onset (*r* = −0.219, *p* = 0.038) and AAO (*r* = −0.216, *p* = 0.041). Neutrophil was negatively correlated with HAMA scores (*r* = −0.526, *p* = 0.046). Monocyte was negatively correlated with UHDRS-TFC (*r* = −0.262, *p* = 0.014). LMR positively was correlated with HAMD scores (*r* = 0.334, *p* = 0.025) (Table 3). In addition, Pearson’s correlation analysis revealed that lymphocyte count (*r* = −0.286, *p* = 0.013), NLR (*r* = 0.295, *p* = 0.011), PLR (*r* = 0.231, *p* = 0.038), SII (*r* = 0.251, *p* = 0.027) were associated with a progression rate of HD (TFC progression rate) (Table 3). However, no associations were found between all the clinical features and the WBC count.

**Table 3:** Association of inflammatory indicators with clinical characteristics in patients with Huntington’s disease (HD).

	Neutrophil (10^9^/L)		Lymphocyte (10^9^/L)		Monocyte (10^9^/L)		LMR		NLR		PLR		SII	
	*r*	*p*-value	*r*	*p*-value	*r*	*p*-value	*r*	*p*-value	*r*	*p*-value	*r*	*p*-value	*r*	*p*-value
Age of onset	0.043	0.696	−0.274	0.009*	−0.017	0.877	0.045	0.675	0.225	0.033*	−0.219	0.038*	−0.024	0.826
Age of diagnosis	0.073	0.499	−0.188	0.074	0.092	0.385	−0.059	0.575	0.209	0.047*	−0.179	0.088	0.009	0.930
AAO	0.073	0.622	−0.227	0.032*	0.006	0.959	0.035	0.743	0.223	0.035*	−0.216	0.041*	−0.025	0.817
UHDRS-IV	−0.042	0.705	−0.075	0.487	−0.262	0.014*	0.068	0.527	−0.074	0.491	0.082	0.444	−0.025	0.816
HAMD	−0.231	0.152	0.042	0.784	−0.186	0.226	0.334	0.025*	−0.122	0.423	0.147	0.336	0.031	0.842
HAMA	−0.526	0.046*	−0.047	0.863	−0.217	0.421	0.218	0.415	−0.352	0.182	0.118	0.662	−0.125	0.646
SDMT	−0.178	0.150	0.186	0.129	0.011	0.927	0.070	0.572	−0.278	0.022*	−0.020	0.869	−0.119	0.336
TFC progression rate	0.092	0.243	−0.286	0.013*	−0.123	0.174	−0.166	0.103	0.295	0.011*	0.231	0.038*	0.251	0.027*

LMR, lymphocyte-to-monocyte ratio; NLR, neutrophil-to-lymphocyte ratio; PLR, platelet-to-lymphocyte ratio; SII, systemic immune-inflammation index; AAO, age of motor symptom onset; HAMD, Hamilton Depression Scale; HAMA, Hamilton Anxiety Scale; TFC, UHDRS-total functional capacity.

^*^p < 0.05.

In the multivariate linear regression analysis, lymphocyte was negatively correlated with the age of onset (β = −0.114, *p* = 0.038). Monocyte negatively correlated with UHDRS-TFC (β = −0.281, *p* = 0.012). Additionally, progression rate of HD (TFC progression rate) was found to be significantly associated with lymphocyte count (β = −0.278, *p* = 0.027), NLR (β = 0.365, *p* = 0.005), PLR (β = 0.315, *p* = 0.012), and SII (β = 0.405, *p* = 0.002) (Table 4).

**Table 4:** Association of inflammatory indicators with clinical characteristics in Huntington’s patients (HD) adjusting for age, sex, and CAG repeat length.

	Neutrophil (10^9^/L)		Lymphocyte (10^9^/L)		Monocyte (10^9^/L)		LMR		NLR		PLR		SII	
	β	*p*-value	β	*p*-value	β	*p*-value	β	*p*-value	β	*p*-value	β	*p*-value	β	*p*-value
Age of onset	−0.051	0.384	−0.114	0.038*	0.041	0.471	−0.068	0.231	0.031	0.577	0.001	0.988	0.008	0.882
Age of diagnosis	−0.043	0.397	−0.023	0.639	0.124	0.010*	−0.112	0.020*	0.011	0.821	0.037	0.441	0.041	0.399
AAO	−0.036	0.531	−0.066	0.234	0.071	0.204	−0.070	0.211	0.041	0.463	−0.149	0.085	0.014	0.804
UHDRS-TFC	−0.014	0.907	−0.095	0.401	−0.281	0.012*	0.111	0.316	0.058	0.604	0.011	0.919	−0.01	0.931
HAMD	−0.244	0.192	0.052	0.754	−0.21	0.236	0.325	0.052	−0.220	0.200	0.180	0.291	0.055	0.752
HAMA	−0.413	0.161	0.007	0.981	−0.249	0.433	0.285	0.376	−0.351	0.193	0.113	0.681	−0.161	0.572
SDMT	−0.061	0.618	0.136	0.255	0.006	0.963	0.093	0.450	−0.152	0.219	−0.098	0.410	−0.093	0.427
TFC progression rate	0.167	0.214	−0.278	0.027*	−0.102	0.431	−0.223	0.087	0.365	0.005*	0.315	0.012*	0.405	0.002*

LMR, lymphocyte-to-monocyte ratio; NLR, neutrophil-to-lymphocyte ratio; PLR, platelet-to-lymphocyte ratio; SII, systemic immune-inflammation index; AAO, age of motor symptom onset; HAMD, Hamilton Depression Scale; HAMA, Hamilton Anxiety Scale; TFC, UHDRS-total functional capacity.

^*^p < 0.05.

### 3.5 Prognosis analysis of inflammatory indicators in patients with HD

**Figure 3:**
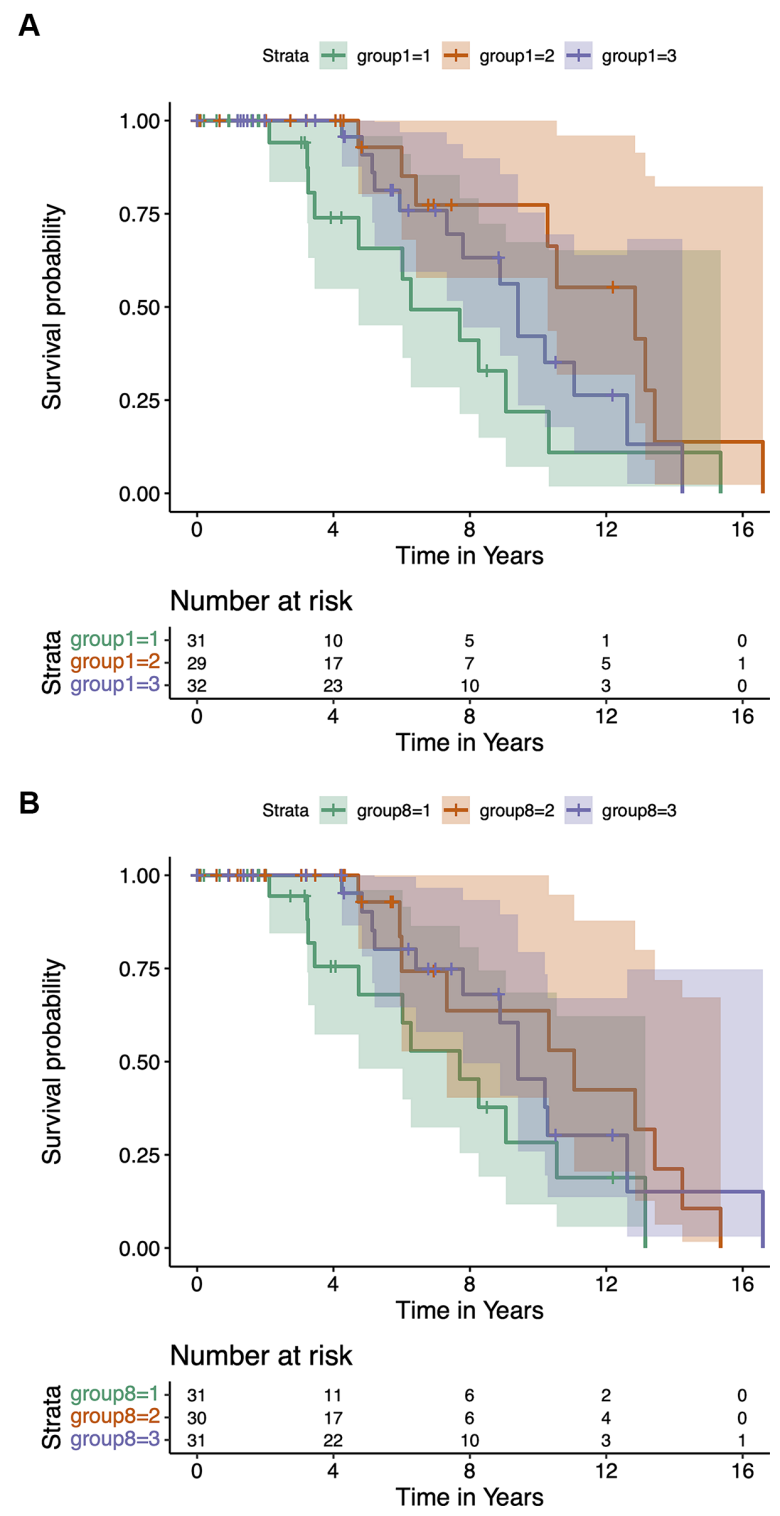
Kaplan–Meier survival analysis of inflammatory profiles in HD patients. Variability in the duration of the loss-of-function state was significant across groups stratified by WBC counts (Breslow *p* = 0.010) **(A)**. Distinct differences in the duration of the loss-of-function state were also observed among monocyte level groups (Breslow *p* = 0.043) **(B)**.

Total of 35 patients including 11 (11.96%) died HD patients and 24 patients (26.09%) with TFC scores of 0–2 at the end of the follow-up period were considered to have an outcome occurring. Our analysis using Kaplan–Meier analysis did not reveal significant differences when categorizing HD patients based on LMR, NLR, PLR, and SII subgroups. As detailed in Supplementary Table S5. Kaplan–Meier analysis presented in Figure 3A showed that the duration of the loss-of-function state was significantly different among the three WBC subgroups (Breslow *p* = 0.010). The duration of loss-of-function state in high WBC subgroup patients was shorter than those in medium WBC subgroup (Breslow *p* = 0.012) and low WBC subgroup (Breslow *p* = 0.034). There was no statistically significant difference in the duration of loss-of-function state between the medium WBC subgroup and the low WBC subgroup (Breslow *p* = 0.232). Furthermore, Figure 3B showed that the duration of loss-of-function state was significantly different among the three monocyte subgroups (Breslow *p* = 0.043). The duration of loss-of-function state in high monocyte subgroup patients was shorter than those in medium monocyte subgroup (Breslow p = 0.043) and low monocyte subgroup (Breslow *p* = 0.053). There was no statistically significant difference in the duration of loss-of-function state between the medium monocyte subgroup and the low monocyte subgroup (Breslow *p* = 0.570).

We further performed a multivariable Cox proportional hazards regression analysis to demonstrate the predictive significance of WBCs and monocytes on the occurrence of loss of function. Higher levels of WBCs (HR = 0.66, 95% CI, 0.42–1.05; *p* = 0.080) and monocytes (HR = 0.63, 95% CI, 0.39–1.01; *p* = 0.056) tend to increase the risk of disability and mortality in HD patients. Univariate Cox proportional hazards regression analysis for survival is shown in Supplementary Table S6. When WBCs and monocytes were stratified into three levels according to their tertiles, after adjusting for age, sex, and CAG repeat length, the hazard ratio for the medium WBC group compared with the high WBC group was 0.35 (HR = 0.35, 95% CI, 0.135–0.912; *p* = 0.032), the hazard ratio for low WBC group compared with high WBC group was 0.469 (HR = 0.469, 95% CI, 0.199–1.104; *p* = 0.083); the hazard ratio for medium monocyte group compared with high monocyte group was 2.506 (HR = 2.506, 95% CI, 1.037–6.053; *p* = 0.041), the hazard ratio for low monocyte group compared with high monocyte group was 0.867 (HR = 0.867, 95% CI, 0.324–2.316; *p* = 0.775). The highest WBCs and monocytes increased the risk of disability and mortality in patients with HD.

## 4 Discussion

As far as we know, this is the first study to explore the relationship between HD and CBC-derived inflammatory indexes and their impact on the progression and prognosis of the disease. Our study found that HD patients had a lower lymphocyte count and a higher neutrophil count compared with HCs. More importantly, the NLR, PLR, and SII were significantly higher in patients with HD, regardless of disease severity. We found that elevated inflammatory status of innate immunity, reflected by increased NLR, PLR and SII, was associated with faster disease progression. Additionally, higher WBCs and monocyte were significantly associated with faster loss of function state in patients with HD. Our results suggest that peripheral inflammation plays a significant role in developing patients with HD.

Consistent with the results in patients with ALS, AD, and PD (11, 19, 32), NLR, PLR, and SII were significantly higher in patients with HD compared with HCs, suggesting neuroinflammation is a pivotal pathological process in neurodegenerative diseases. NLR, PLR, and SII are commonly used and cost-effective biomarkers that reflect the balance between innate (neutrophil) and adaptive (lymphocyte) immune responses reported in various pathological conditions (11, 31). We also observed other aberrant inflammatory biomarkers, namely decreased absolute lymphocyte count and decreased LMR, in HD patients, which previously reported in PD and ALS (18, 31). Recently, von Essen MR et al. reported that in *HTT* gene expansion carriers, there are higher levels of proinflammatory cytokines such as IL-17 in the cerebrospinal fluid (CSF) before the motor onset of HD (33). In the meantime, there was an increased prevalence of IL-17-producing Th17.1 cells in the CSF. The frequency of intrathecal Th17.1 cells is negatively correlated with the progression of HD, potentially playing a role in the early stages of the disease. The imbalance between proinflammatory and regulatory T cells could lead to a proinflammatory environment in the CSF of HTT gene expansion carriers (33). Therefore, the lower number of lymphocytes may reflect migration of T-cells into the central nervous system (34). Additionally, the correlation between a reduction of total lymphocytes and a faster disease progression could indicate that the lymphocytes’ protective immune function might be compromised in HD patients, as reported in PD (35). However, higher lymphocytes have a lower age of onset. We propose that while higher lymphocyte counts correlate with an earlier age of onset, suggesting a potential role in disease initiation, they are also linked to slower progression rates, indicating a possible protective immune response in the later stages of HD. This dual role of lymphocytes may reflect complex, stage-dependent immune dynamics in HD, which could be indicative of different inflammatory mechanisms operating at various points in the disease’s trajectory. Additionally, guidance from Chinese consensus on HD suggests that adult-onset patients with an earlier onset tend to have a longer disease duration, while elder-onset patients with a shorter disease duration and faster progression (36). Further research is necessary to unravel these distinct inflammatory patterns and understand their implications fully.

Neutrophils are essential contributors to chronic inflammation (37). The primary roles of neutrophils encompass phagocytosis, degranulation, and secreting neutrophil extracellular traps (38). Neutrophil extracellular traps can disrupt the blood–brain barrier, either directly through histones and various proteases or indirectly by enhancing type I interferon responses (39). Neutrophil degranulation results in the liberation of a variety of enzymes, including myeloperoxidase (MPO), neutrophil elastase (NE), and matrix metalloproteinases (MMPs) (40). Previous studies have confirmed a significant increase in the MPO/WBC ratio in HD patients compared to HCs, suggesting an increase in WBC activity (41). Our study found a trend toward an increased absolute neutrophil count in HD patients. Understanding and mitigating neuroinflammation is therefore crucial for developing therapeutic interventions for these debilitating conditions.

Within the HD group, we did not found differences in the NLR, PLR, LMR, and SII according to sex, age of onset, disease severity (based on TFC score), and BMI. Furthermore, we found no correlation between these inflammatory markers and UHDRS scores. This observation is consistent with similar studies in PD (18, 32), where IL-6 was shown to increase significantly with disease progression, particularly from pre-manifest to manifest stages and from early to moderate stages (42). In contrast, significant correlations were identified between plasma IL-6 levels and both UHDRS-TFC and TMS scores (42). The absence of studies examining the relationship between NLR, SII, and other inflammatory markers underscores the novelty of our findings. Our study highlighted that the TFC progression rate has a positive correlation between NLR, PLR, and SII and is negatively correlated with lymphocyte count. Previous study reported that patients with HD exhibit dysfunction in their immune systems both centrally, through the activation of microglial cells (6, 43, 44), and peripherally, where detectable elevations of proinflammatory mediators occur before the onset of the disease (45), suggesting that the presence of mHTT results in a global immune response, with central pathology being reflected peripherally (46). Analysis of monocyte transcriptomes from HD patients shows proinflammatory phenotype, reflecting mutant HTT priming effect (47). Peripheral blood mononuclear cells in individuals with HD are likely responsible for the increased production of pro-inflammatory cytokines. Research has shown that monocytes and macrophages isolated from patients with HD produce significantly more IL-6, IL-8, and TNFα when stimulated with lipopolysaccharide compared to individuals without the disease (48). In HD mouse models, macrophages and monocytes that promote immune hyperactivation during disease progression (49). Moreover, platelets contain several essential neurotransmitters, including GABA, glutamate, serotonin, epinephrine, dopamine, and histamine, that help in intercellular communication between brain cells (50). The platelets of patients with HD exhibit various abnormalities, including aberrant amplification of adenosine A receptor (A2AR) signaling. As the A2AR is expressed in GABA/enkephalin spiny neurons, it may play a role in the pathogenesis of HD (50). Platelets play a role in the permeability of the blood–brain barrier in HD, suggesting their potential contribution to the pathogenesis of the disease (51). Platelets release serotonin upon encountering particular glycolipid structures on neurons and astrocytes following the disruption of the blood–brain barrier, resulting in neuroinflammation in neurodegeneration (32). In our study, we observed a positive correlation between PLR and the TFC progression rate, suggesting that inflammation tends to escalate with the advancement of the disease.

Therefore, a single marker may not be enough to capture the overall peripheral immune dysregulation and inflammatory status in individuals with HD. Our study yielded intriguing findings. Firstly, we observed significant correlations between various inflammatory indicators and key clinical aspects of HD. The absolute number of monocyte negative correlations were detected against TFC (the worse the ability to live independently, the higher the level of circulating monocytes). The multivariable Cox regression analysis showed that a higher WBC count and monocyte counts were significantly correlated with an increased risk of disability and mortality among patients with HD. Elevated total WBC count and monocyte count have been reported to be a significant indicator of reduced survival in patients with cardiovascular disease (52) and cancer (53), showing a positive correlation with all-cause mortality (54). This is the first study to demonstrate an association between higher WBC count and monocyte and HD prognosis.

One of the strengths of this study is the longtime of follow-up, and the consistency of measurements was increased using the same clinical laboratory. Our study had some limitations that need to be considered. First, we are unable to capture the effect of lymphocyte subpopulations. Second, we did not include other peripheral inflammatory biomarkers in our analysis. Third, confounding factors may have influenced observed CBC counts as the present study was an observational study. Fourth, we only evaluated the correlation between baseline inflammation markers and survival in patients with HD. Hence, a longitudinal study that investigates inflammation markers at different time points is essential to assess their contribution to the advancement of HD. We plan to investigate this matter in an upcoming cohort study to examine potential alterations in inflammation markers throughout the progression of the disease.

## 5 Conclusion

This study confirmed the presence of immune system abnormalities in patients with HD and explored the correlation between peripheral immunity and the clinical characteristics of HD. We demonstrated that the inflammatory status of innate immunity may speed up disease progression. Peripheral immune dysfunction in HD is likely to be mediated primarily by the innate rather than the adaptive immune system. Higher WBCs and monocytes were significantly associated with higher disability and mortality in patients with HD. Different peripheral immune cells may specifically contribute to the multifaceted mechanisms and clinical features in HD. In conclusion, the study gives evidence of the involvement of peripheral immunity in the HD progression and possible association with the prognosis. Understanding and mitigating neuroinflammation is therefore crucial for developing therapeutic interventions for these debilitating conditions. Further studies are necessary to transform our findings into innovative clinical markers and potential therapeutic targets.

## Data availability statement

The raw data supporting the conclusions of this article will be made available by the authors, without undue reservation.

## Ethics statement

The studies involving humans were approved by the institutional ethics committee of Sichuan University (approval number 2015-236). The studies were conducted in accordance with the local legislation and institutional requirements. The participants provided their written informed consent to participate in this study.

## Author contributions

J-QX: Investigation, Writing – original draft, Writing – review & editing, Methodology. Y-FC: Data curation, Formal analysis, Funding acquisition, Investigation, Methodology, Validation, Writing – original draft, Writing – review & editing. S-RZ: Formal analysis, Investigation, Methodology, Validation, Writing – review & editing. Y-ZM: Formal analysis, Investigation, Validation, Writing – review & editing. J-JF: Formal analysis, Investigation, Methodology, Writing – review & editing. T-MY: Investigation, Writing – review & editing. L-YZ: Data curation, Methodology, Writing – review & editing. J-MB: Conceptualization, Project administration, Writing – review & editing. H-FS: Conceptualization, Funding acquisition, Project administration, Supervision, Writing – review & editing.
