# Supplementary material for: The characteristic and prognostic role of blood inflammatory markers in patients with Huntington’s disease from China
Source: Front Neurol. 2024 Mar 26;15:1374365. doi: 10.3389/fneur.2024.1374365 (PMC11002148; doi:10.3389/fneur.2024.1374365)
Supplement: Supplementary file 1 [file Data_Sheet_1.docx]

TableS1. Peripheral inflammatory indicators in patients with Huntington's disease (HD) and healthy controls (HCs) across different sex subgroups.

|  | HD | | P  value | HC | | P  value | P  value |
| --- | --- | --- | --- | --- | --- | --- | --- |
|  | Male  (n=35) | Female  (n=57) | Male *v.s.* Female | Male  (n=36) | Female  (n=56) | Male  HD *v.s.* HC | Female  HD *v.s.* HC |
| RBCs (10^12^/L) | 4.86±0.49 | 4.30±0.33 | <0.0001* | 4.95±0.37 | 4.48±0.26 | 0.364 | 0.002* |
| Hemoglobin (g/L) | 146.60±18.17 | 126.4±11.04 | <0.0001* | 154.9±12.24 | 135.2±8.52 | 0.047* | <0.001* |
| Platelet(10^9^/L) | 201.10±74.72 | 204.1±72.13 | 0.861 | 187.7±51.91 | 202.9±57.32 | 0.384 | 0.927 |
| WBCs (10^9^/L) | 5.91±0.98 | 5.18±1.38 | 0.007* | 5.93±1.59 | 5.50±1.19 | 0.886 | 0.198 |
| Neutrophils(10^9^/L) | 3.58±1.06 | 3.03±0.91 | 0.009* | 3.31±0.99 | 3.08±0.84 | 0.287 | 0.762 |
| Lymphocytes(10^9^/L) | 1.79±0.48 | 1.61±0.46 | 0.059 | 1.90±0.58 | 1.89±0.59 | 0.387 | 0.005* |
| Monocyte (10^9^/L) | 0.39±0.13 | 0.33±0.12 | 0.027* | 0.39±0.13 | 0.32±0.11 | 0.972 | 0.698 |
| Eosinophilia (10^9^/L) | 0.12±0.07 | 0.10±0.06 | 0.237 | 0.15±0.09 | 0.09±0.04 | 0.167 | 0.257 |
| Basophilia (10^9^/L) | 0.02±0.01 | 0.02±0.01 | 0.686 | 0.03±0.02 | 0.03±0.01 | 0.162 | 0.006* |
| LMR | 4.84±1.63 | 5.30±1.70 | 0.386 | 5.27±1.83 | 6.14±2.22 | 0.301 | 0.254 |
| NLR | 2.37±1.16 | 2.01±0.67 | 0.056 | 1.84±0.61 | 1.67±0.61 | 0.020* | 0.007* |
| PLR | 117.80±53.18 | 133.0±50.19 | 0.193 | 105.4±36.72 | 114.6±48.74 | 0.259 | 0.052 |
| SII | 455.70±254.30 | 399.30±147.50 | 0.147 | 357.4±164.6 | 327.4±143.1 | 0.058 | 0.011* |

HC, healthy control; RBCs, red blood cells; WBC*s*, white blood cells; LMR, lymphocyte-to-monocyte ratio; NLR, neutrophil-to-lymphocyte ratio; PLR, platelet-to-lymphocyte ratio; SII, systemic immune-inflammation index

* *p* < 0.05

*P* value compared from male and female patients with HD was adjusted by age, and disease severity(UHDRS_TFC)

TableS2. Peripheral inflammatory indicators in patients with Huntington's disease (HD) between high and low BMI groups.

|  | HD | | P  value |
| --- | --- | --- | --- |
|  | BMI_high(n=19) | BMI_low(n=25) | BMI_low *v.s.* BMI_high |
| RBCs (10^12^/L) | 4.49±0.49 | 4.50±0.37 | 0.865 |
| Hemoglobin (g/L) | 132.60±19.72 | 134.60±14.37 | 0.477 |
| Platelet(10^9^/L) | 219.73±73.78 | 212.76±54.53 | 0.566 |
| WBCs (10^9^/L) | 5.82±1.34 | 4.96±1.27 | 0.049* |
| Neutrophils(10^9^/L) | 3.19±0.75 | 2.84±0.89 | 0.258 |
| Lymphocytes(10^9^/L) | 1.82±0.46 | 1.65±0.49 | 0.271 |
| Monocyte (10^9^/L) | 0.41±0.14 | 0.36±0.14 | 0.340 |
| Eosinophilia (10^9^/L) | 0.11±0.08 | 0.09±0.05 | 0.648 |
| Basophilia (10^9^/L) | 0.02±0.01 | 0.03±0.01 | 0.306 |
| LMR | 5.09±1.85 | 4.69±1.33 | 0.433 |
| NLR | 1.97±0.80 | 2.07±1.94 | 0.703 |
| PLR | 124.31±70.01 | 137.79±45.64 | 0.591 |
| SII | 443.37±238.11 | 403.47±176.11 | 0.509 |

BMI ,body mass index;RBCs, red blood cells; WBC*s*, white blood cells; LMR, lymphocyte-to-monocyte ratio; NLR, neutrophil-to-lymphocyte ratio; PLR, platelet-to-lymphocyte ratio; SII, systemic immune-inflammation index

* *p* < 0.05

*P* values was adjusted by sex and age

TableS3. Peripheral inflammatory indicators in patients with Huntington's disease (HD) among different age of onset groups.

|  | HD | |  | P  value |
| --- | --- | --- | --- | --- |
|  | age of onset <40 (n=31) | age of onset [40,50] (n=37) | age of onset >50 (n=22) |  |
| RBCs (10^12^/L) | 4.52±0.51 | 4.49±0.44 | 4.49±0.55 | 0.454 |
| Hemoglobin (g/L) | 133.32±20.93 | 134.16±15.05 | 134.36±16.22 | 0.858 |
| Platelet(10^9^/L) | 233.52±80.81 | 195.47±63.19 | 165.77±56.92 | 0.003* |
| WBCs (10^9^/L) | 5.40±1.23 | 5.55±1.32 | 5.24±1.38 | 0.368 |
| Neutrophils(10^9^/L) | 3.13±0.95 | 3.33±0.94 | 3.23±1.22 | 0.565 |
| Lymphocytes(10^9^/L) | 1.76±0.48 | 1.62±0.45 | 1.56±0.47 | 0.222 |
| Monocyte (10^9^/L) | 0.36±0.12 | 0.35±0.11 | 0.33±0.14 | 0.029* |
| Eosinophilia (10^9^/L) | 0.09±0.07 | 0.11±0.07 | 0.11±0.07 | 0.309 |
| Basophilia (10^9^/L) | 0.02±0.012 | 0.02±0.013 | 0.02±0.011 | 0.494 |
| LMR | 5.16±2.01 | 4.91±1.17 | 5.31±1.67 | 0.473 |
| NLR | 1.96±0.88 | 2.31±0.90 | 2.19±0.93 | 0.288 |
| PLR | 139.62±54.10 | 128.16±49.73 | 109.24±50.26 | 0.157 |
| SII | 430.09±204.54 | 440.46±189.94 | 378.48±200.69 | 0.283 |

RBCs, red blood cells; WBC*s*, white blood cells; LMR, lymphocyte-to-monocyte ratio; NLR, neutrophil-to-lymphocyte ratio; PLR, platelet-to-lymphocyte ratio; SII, systemic immune-inflammation index

* *p* < 0.05

*P* values was conducted by ANCOVA analysis, and adjusted by sex and CAG repeat.

Table S4. Clinical characteristics of patients with Huntington’s disease (HD).

|  | HD  (n=92) | HD subgroups | | | *p* value among stages |
| --- | --- | --- | --- | --- | --- |
|  |  | Stage I (n=21) | Stage II (n=39) | Stage III-V (n=29) |  |
| Age (years) | 54.27±11.36 | 53.91±10.72 | 54.22±11.59 | 56.98±10.93 | 0.525 |
| Sex (male, %) | 38.04% (35/92) | 33.33% (7/21) | 38.40% (15/39) | 41.30% (12/29) | 0.845 |
| BMI (kg/m^2^) | 20.93±2.64 | 21.92±1.66 | 20.63±2.220 | 21.47±3.48 | 0.470 |
| Education (years) | 9.13±4.45 | 9.62±4.61 | 9.59±4.56 | 7.64±4.65 | 0.184 |
| CAG repeat number | 44 (42-46) | 44(42-45.5) | 44(42-48) | 42(42-45) | 0.275 |
| CAP | 489.2±118.0 | 457.8±108.7 | 501.4±106.8 | 491.0±131.8 | 0.379 |
| Family history | (73.81%)84/62 | (72.22%)18/13 | (79.49%)39/31 | (68.0%)25/17 | 0.586 |
| Paternal inheritance | (61.29%)62/38 | (53.85%)13/7 | (70.97%)31/22 | (47.06%)17/8 | 0.181 |
| Genetic anticipation | (39.76%)83/33 | (44.44%)18/8 | (41.0%) (39/16) | (33.3%) 24/8 | 0.802 |
| Age of onset(years) | 42.67±10.04 | 42.64±9.49 | 42.77± 9.23 | 44.28±10.69 | 0.786 |
| Age of diagnosis | 47.05±10.81 | 45.50±8.66 | 47.19±11.08 | 50.46±11.36 | 0.239 |
| HD duration(years) | 5.41±3.93 | 3.57±1.90 | 5.17±3.27 | 7.64±4.49 | 0.001* |
| DOD (years) | 4.17±3.61 | 2.96±2.01 | 3.84±3.17 | 5.91±4.04 | 0.004* |
| AAO (years) | 43.14±10.33 | 42.64±9.49 | 43.73±9.91 | 44.48±10.67 | 0.819 |
| UHDRS-I | 38.18±15.76 | 26.81±10.23 | 37.05±11.87 | 48.14±17.19 | <0.001* |
| UHDRS-II | 17.77±5.49 | 22.24±2.17 | 18.92±3.0 | 12.86±5.68 | <0.001* |
| UHDRS-III | 82.43±16.14 | 96.43±5.04 | 85.38±8.91 | 66.90±15.89 | <0.001* |
| UHDRS-IV | 8.13±3.31 | 12.05±0.86 | 8.54±1.12 | 4.14±1.48 | <0.001* |
| HAMD (scores) | 11.17±6.98 | 8.88±7.36 | 11.40±6.78 | 12.56±7.08 | 0.482 |
| HAMA (scores) | 8.25±4.16 | 8.67±8.08 | 7.50±4.80 | 8.50±2.83 | 0.927 |
| BDI (scores) | 6.98±5.89 | 6.67±4.89 | 8.87±7.36 | 6.27±5.08 | 0.544 |
| PBA-s (scores) | 13.61±13.47 | 3.27±2.76 | 15.78±11.04 | 14.52±13.83 | 0.001* |
| ***Cognitive tests*** |  |  |  |  |  |
| MMSE (scores) | 21.61±5.81 | 23.83±5.13 | 24.07±3.46 | 18.04±5.13 | <0.001* |
| VFT-letter | 9.59±5.76 | 11.61±4.87 | 10.76±5.08 | 6.68±4.82 | 0.006* |
| VFT-animal | 10.24±6.09 | 10.00±3.24 | 10.13±3.09 | 7.29±1.70 | 0.099 |
| SDMT | 15.42±11.20 | 22.61±12.02 | 15.44±7.22 | 4.82±5.88 | <0.001* |
| Total Stroop | 85.47±41.37 | 109.3±45.63 | 84.97±29.01 | 48.20±36.42 | <0.001* |
| Trail test A | 131.9±62.33 | 58.00±11.22 | 122.5±47.50 | 177.1±59.02 | 0.001* |
| Trail test B | 182.5±60.92 | 119.8±40.70 | 197.1±50.04 | 240.0±0.00 | 0.001* |

BMI ,body mass index ;CAP,CAG-Age Product score; DOD, diagnostic of delay; AAO, age of motor symptom onset ; UHDRS , Unified Huntington's Disease Rating Scale ; HAMD, Hamilton Depression Scale; HAMA ,Hamilton Anxiety Scale; BDI, Beck Depression Inventory; PBA‐s, short version of problem behavior assessment; MMSE, mini–mental state examination ; VFT, verbal fluency test; SDMT, symbol digit modality test

* *p* < 0.05

Table S5. Kaplan–Meier analysis of subgroups of peripheral inflammatory indicators.

|  | Estimated survival time(Year) (Std. Error) | | | P  value |
| --- | --- | --- | --- | --- |
|  | low | medium | high |  |
| RBCs (10^12^/L) | 7.80±2.08 | 8.89±2.41 | 10.20±1.24 | 0.442 |
| Hemoglobin (g/L) | 10.28±1.75 | 7.32±1.27 | 9.41±1.82 | 0.316 |
| Platelet(10^9^/L) | 9.06±0.83 | 9.41±2.06 | 10.20±1.65 | 0.927 |
| WBCs (10^9^/L) | 9.41±0.47 | 12.85±2.78 | 6.27±1.41 | 0.01* |
| Neutrophils(10^9^/L) | 9.06±1.52 | 10.55±2.38 | 9.41±1.37 | 0.916 |
| Lymphocytes(10^9^/L) | 7.80±0.43 | 7.32±1.86 | 11.06±1.61 | 0.214 |
| Monocyte (10^9^/L) | 7.70±1.31 | 11.06±2.77 | 9.41±0.75 | 0.043* |
| Eosinophilia (10^9^/L) | 10.28±1.30 | 8.26±1.16 | 10.20±5.23 | 0.561 |
| Basophilia (10^9^/L) | 9.41±0.92 | 10.20±4.97 | 8.89±1.84 | 0.619 |
| LMR | 7.80±1.42 | 10.20±0.90 | 9.41±2.54 | 0.229 |
| NLR | 10.32±3.30 | 9.06±0.36 | 7.80±1.21 | 0.513 |
| PLR | 9.41±2.11 | 9.06±0.41 | 10.28±2.55 | 0.580 |
| SII | 9.41±0.94 | 8.89±2.23 | 9.41±2.00 | 0.716 |

RBCs, red blood cells; WBC*s*, white blood cells; LMR, lymphocyte-to-monocyte ratio; NLR, neutrophil-to-lymphocyte ratio; PLR, platelet-to-lymphocyte ratio; SII, systemic immune-inflammation index

* *p* < 0.05

Table S6. Univariate Cox proportional-hazards regression analyses for survival of peripheral inflammatory indicators.

| Hematological test index | HR (95% CI) | P value |
| --- | --- | --- |
| RBCs (10^12^/L) | 1.01(0.60-1.69) | 0.982 |
| Hemoglobin (g/L) | 0.99(0.54-1.83) | 0.981 |
| Platelet(10^9^/L) | 1.20(0.74-1.94） | 0.456 |
| WBCs (10^9^/L) | 0.66(0.42-1.05) | 0.080 |
| Neutrophils(10^9^/L) | 0.90(0.56-1.45) | 0.659 |
| Lymphocytes(10^9^/L) | 0.82 (0.51-1.32) | 0.422 |
| Monocyte (10^9^/L) | 0.63 (0.39-1.01) | 0.056 |
| Eosinophilia (10^9^/L) | 1.05 (0.64-1.73) | 0.833 |
| Basophilia (10^9^/L) | 1.08 (0.71-1.62) | 0.726 |
| LMR | 0.80(0.47-1.35) | 0.404 |
| NLR | 1.46(0.84-2.53) | 0.180 |
| PLR | 1.38(0.89-2.15) | 0.148 |
| SII | 0.96(0.60-1.53) | 0.867 |

CI, confidence interval; HR, hazard ratio; RBCs, red blood cells; WBC*s*, white blood cells; LMR, lymphocyte-to-monocyte ratio; NLR, neutrophil-to-lymphocyte ratio; PLR, platelet-to-lymphocyte ratio; SII, systemic immune-inflammation index

* *p* < 0.05
